# Expression of Ferroptosis-Related Proteins Glutathione Peroxidase 4, Nuclear Factor Erythroid 2-Related Factor 2, and Solute Carrier Family 7 Member 11 in Gastric Cancer Patients

**DOI:** 10.5152/tjg.2023.22670

**Published:** 2023-12-01

**Authors:** Yitian Sun, Zhiyu Zhu, Ting Duan, Guoxiong Li

**Affiliations:** 1 The Affiliated Hospital, Hangzhou Normal University, Hangzhou, Zhejiang, China; 2 Department of Medical Oncology, The Affiliated Hospital, Hangzhou Normal University Faculty of Pharmacy, Hangzhou, Zhejiang, China

**Keywords:** Gastric cancer, ferroptosis, GPX4, NRF2, SLC7A11

## Abstract

**Background/Aims:**

The aim of this study was to investigate the expression of ferroptosis-related targets glutathione peroxidase 4, nuclear factor erythroid 2-related factor 2, and solute carrier family 7 member 11 in gastric cancer and the correlation between their expression and the clinicopathological characteristics and prognosis of gastric cancer patients.

**Materials and Methods:**

Forty-two gastric cancer samples and paracancerous samples were included, and all cases were detected with glutathione peroxidase 4, nuclear factor erythroid 2-related factor 2, and solute carrier family 7 member 11 by immunohistochemistry. Six gastric cancer cell lines and 1 normal gastric epithelial cell were stably cultured, and the expression of target genes of different cell lines was detected using western blot and polymerase chain reaction. Public data were downloaded from the database, and analyses were performed by software including Statistical Package for the Social Sciences and Prism.

**Results:**

A high glutathione peroxidase 4 expression level was found in 7 (16.67%) cancer tissues and 0 (0.00%) paracancerous tissues (*P* = .012). Nuclear factor erythroid 2-related factor 2 was upregulated in 23 (54.76%) cancer tissues and 2 (4.76%) paracancerous tissues (*P* < .001). Solute carrier family 7 member 11 showed increased expression in 4 (9.52%) cancer tissues and 1 (2.38%) paracancerous tissue (*P* = .356). No significant association existed between their expression and the clinicopathological characteristics and prognosis of gastric cancer patients. Glutathione peroxidase 4 and nuclear factor erythroid 2-related factor 2 expressions were higher in all 6 gastric cancer cell lines compared to normal gastric epithelial cells.

**Conclusion:**

Glutathione peroxidase 4 and nuclear factor erythroid 2-related factor 2 expressions were significantly higher in gastric cancer, which may be potential biomarkers of gastric cancer.

**Main Points**

- Glutathione peroxidase 4 (GPX4) and nuclear factor erythroid 2-related factor 2 (NRF2) were upregulated in gastric cancer (GC) compared to normal gastric tissues.
- There was no significant association between GPX4, NRF2, and solute carrier family 7 member 11 expressions and the clinicopathological characteristics of the patients.
- The high expression of GPX4 may act as a poor prognostic marker of GC patients.
- Specific ferroptosis-related proteins may be potential novel biomarkers of GC.

## Introduction

Gastric cancer (GC) is one of the most common malignant tumors in the gastrointestinal system. It was estimated that nearly 1 million cases of GC were diagnosed, leading to more than 730 000 deaths.^1^ Early symptoms of GC are insidious, and patients are usually diagnosed at the advanced stage. Although significant progress has been made in early diagnosis with the development of endoscopic technology, the prognosis of patients is still not optimistic with high recurrence and mortality rate.^2^ Therefore, it is particularly important to find biomarkers that can be useful for early diagnosis and prognosis of GC.

In normal condition, the body maintains relatively balanced redox homeostasis, which is essential for preventing oxidative damage to biological molecules such as DNA, proteins, and lipids. Tumor cells show complex metabolic adaptation strategies, which help them to survive and grow even under metabolic stress conditions.^3^ Ferroptosis is an example of “regulated cell death,” which is being explored as an alternative method to eradicate antiapoptotic cancer cells in recent years. Intracellular redox homeostasis can affect ferroptosis through diverse pathways including nicotinamide adenine dinucleotide phosphate (NADP(+))/reduced NADP(+) (NADPH) andglutathione (GSH)/oxidized glutathione (GSSG) couple.^4^ GSH-dependent lipid peroxide repairing systems are the first line of defense in protecting cells from oxidative stress damage, and glutathione peroxidase 4 (GPX4) consumes 2 molecules of glutathione and catalyzes the conversion of endogenous lipid peroxides into a harmless substance in the presence of glutathione, thus becoming one of the inhibitory factors of ferroptosis. Nuclear factor erythroid 2-related factor 2 (NRF2), a member of the Cap-N-collar transcription factors family, is a key factor in the development of antioxidant enzymes, and its aberrant expression was detected in a number of cancer tissues. Solute carrier family 7 member 11 (SLC7A11) is an upstream node molecule of glutathione-dependent lipid peroxide repair system, which protects cells from oxidative stress. Here, we retrospectively detected the expression of GPX4, NRF2, and SLC7A11 in GC and further analyzed the relationship between their expressions and clinicopathological parameters and patient prognosis.

## Materials and Methods

### Patient and Tissue Samples

All patient samples and relevant data were collected from the Affiliated Hospital of Hangzhou Normal University, Hangzhou, Zhejiang, China. Forty-two GC patients were collected retrospectively. All cases had undergone surgery (total or partial gastrectomy) in the Department of Gastrointestinal Hepatobiliary Surgery during January 2018 to December 2020, and information about histology, grade, and stage was confirmed by the Department of Pathology. Tumor staging and grading was based on the 8th edition of the American Joint Committee on Cancer staging system. Samples of patients with other malignant tumors or who had received any other anti-cancer therapies (radiotherapy, chemotherapy, or chemoradiotherapy) were excluded. Clinical data were selected through the Electronic Medical Record System. Information about survival and progression was followed from the clinical department by December 2021. The study protocol conforms to the ethical guidelines of the 1975 Declaration of Helsinki (6th revision, 2008), as reflected in a priori approval by the institution’s human research committee. Ethical approval for the research was provided by the Ethics Committee of the Affiliated Hospital of Hangzhou Normal University (number: 2020(E2)-KS-011). The informed consent process was not required for our study in China because our research is a retrospective analysis from paraffin sections but not a perspective study.

### Immunohistochemistry and Staining Evaluation

**Figure 1.**
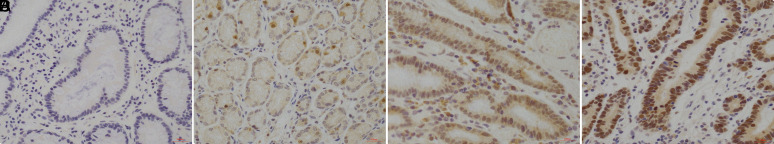
Immunohistochemistry staining intensity according to a 4-point system (original magnification: 200×). Score: 0, negative (A); 1, weak (B); 2, moderate (C); and 3, high (D).

The collected sections were deparaffinized by xylene (X820585, Macklin, Shanghai, China) and rehydrated by descending grades of ethanol (E809056, Macklin, Shanghai, China). Antigen thermal repair was completed in sodium citrate (C1031, Solarbio, Beijing, China), endogenous peroxidase was quenched with hydrogen peroxide, and goat serum was used to reduce nonspecific staining. The specimens were separately incubated with GPX4 (1:250 dilution), NRF2 (1:500 dilution), and SLC7A11 (1:250 dilution), respectively, overnight at 4^°^C. The specimens were then incubated a second time with anti-rabbit antibody [immunoglobulin G (IgG)]. A 3,3'-diaminobenzidine tetrahydrochloride (DAB) Kit (E-IR-R213, Elabscience, Wuhan, China) was applied to detected immune-stain, and counterstain was performed by hematoxylin. For comparison, puncture samples from normal gastric mucosa were served as contrasts for each antibody, and the negative control was achieved using phosphate-buffered saline instead of primary antibodies. Images were obtained with the Leica inverted microscope (Leica DM 4000, Leica, Weztlar, DE). Positive staining cells were counted in 5 random representative areas (200×) and the immunostaining pattern was divided into a 4-point system (0, negative; 1, weak; 2, moderate; and 3, intense) based on the proportion of positive cells.^5^ Representative immunostaining pattern of 4-point scoring is shown in Figure 1. Samples were then classified into 2 groups: the low-expression group (0-1) or the high-expression group (2-3). All staining scores were evaluated by 2 pathologists independently with senior titles and were blinded to clinical data.

### Cell Lines and Culture Methods

The human GC lines AGS, BGC-823, HGC-27, MKN-45, MNK-28, and SGC-7901 were purchased from the National Collection of Authenticated Cell Cultures (STR Authentication, Shanghai, China), and the human normal gastric epithelial cell line GES-1 was obtained from Aoyin Biotechnology Co., Ltd (STR Authentication). Cell lines were cultured in Roswell Park Memorial Institute 1640 medium (#L210KJ, BasalMedia, Shanghai, China) and DMEM (#L110KJ, BasalMedia, Shanghai, China) containing 10% fetal bovine serum (184590, Corille, Australia), 100 units/mL penicillin, and 100 µg/mL streptomycin at 37°C in a humidified environment of 95% air and 5% carbon dioxide. Image of the cell lines were obtained with the Zeiss inverted microscope Primovert (Carl Zeiss, Suzhou, China).

### Antibodies and Reagents

Anti-GPX4 (ab125066), anti-NRF2 (ab89443), and anti-SLC7A11 (ab37185) were purchased from Abcam, Shanghai, China, and anti-Glyceraldehyde-3-phosphate dehydrogenase (GAPDH) (#2118) was obtained from Cell Signaling Technology (CST, Shanghai, China). Anti-rabbit IgG, anti-mouse IgG, and horseradish peroxidase (HRP)-linked antibody (#7074, #7076) were also purchased from CST. Primer sequences were customized from Tsingke Biotechnology Co., Ltd (Shanghai, China).

### Western Blot

Collecting cells and using the radio immunoprecipitation assay (RIPA) buffer (P0013B, Beyotime, Shanghai, China) in the presence of a phenylmethyl sulfonylfluoride (#8553, CST). Protein concentration were determined by the BCA Protein Assay Kit (#P0009, Beyotime, China). Equivalent amounts of the proteins (40 μg/line) were separated on Tris-Tricine Ready Gel (#1611210, Bio-Rad, Hercules, Calif, USA) sodium dodecyl sulfate-polyacrylamide gel electrophoresis (SDS-PAGE) for nitrocellulose membrane polyvinylidene fluoride (PVDF) (ISEQ00010, Merck Millipore, Massachusetts, USA) blotting. The blotted membranes were blocked with 5% skim milk (#P0216-300g, Beyotime, Shanghai, China) for 1 hour at ambient temperature and incubated with primary antibodies overnight at 4^◦^C. They were washed with tris-buffered saline Tween-20 (TBST) (CW0043S, CWBIO, Jiangsu, China), and the immunoreactive bands were visualized by enhanced chemiluminescence reagent. All bands were analyzed by NIH image 1.62. All experiments were conducted in triplicate.

### Real-Time Quantitative Polymerase Chain Reaction

Total RNA was extracted by Trizol reagent (#AG21102, Accurate Biology, Hunan, China). One thousand nanograms of mRNA was used to synthesize complementary DNA (cDNA) for each sample using Prime-ScriptTM II Reverse Transcriptase (#R323-01, Vazyme, Nanjing, China) and polymerase chain reaction (PCR) Amplifier (#T100 Thermal Cycler, Bio-Rad). Complementary DNA concentration was determined using Microvolume UV-Vis Spectrophotometer (NanoDrop One, Thermo Fisher, Waltham, USA). Primer sequences for real-time quantitative polymerase chain reaction (RT-qPCR) are shown in Table 1, and GAPDH was used as the endogenous control for mRNA. Polymerase chain reactions were performed in a total volume of 10 µL, which included 5 µL synergy brands (SYBR) reagent (#Q711-02, Vazyme, Nanjing, China) and 0.5 µL cDNA together with primers at the concentration of 0.25 mM. For the detection of gene expression, CFX96 Touch PCR Detection System (Bio-Rad) was used for PCR. All results were computed by the 2^−ΔΔCt^ method for the relative quantification of target mRNAs in GC cells and normal controls. Each detection was established in 4 duplicates and conducted for 3 times repeatedly.

### Statistical Analyses and Data Retrieval

Statistical analyses were performed by Statistical Package for the Social Sciences (SPSS) 26.0 statistical software package (IBM corp., Armonk, NY USA) and GraphPad Prism 9.0 (GraphPad Software Inc., San Diego, Calif, USA). Quantitative data are presented as the mean ± SD and categorical variables are described using their absolute frequencies. The 2-tailed *χ* ^2^ test and rank-sum test were used to analyze the association of protein expression and clinicopathological parameters. A survival curve was generated using the Kaplan–Meier method and assessed with the log-rank test. The information and expression of 3 target proteins were analyzed by searching Gene Expression Profiling Interactive Analysis (GEPIA) databases. The mRNA level of GPX4, NRF2, and SLC7A11 in GC tissues and normal gastric tissues was analyzed with the “Boxplot” option. Survival curve was formed with the “Survival Plots” option. *P* < .05 was considered statistically significant, and highly significant difference was present if *P* ≤ .001.

## Results

### Expression of Glutathione Peroxidase 4, Nuclear Factor Erythroid 2-Related Factor 2, and Solute Carrier Family 7 Member 11 in Gastric Cancer and Paracancerous Tissues

**Figure 2.**
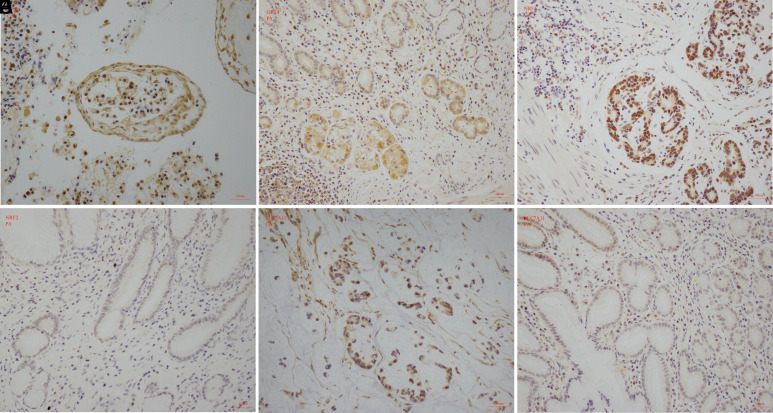
Representative immunohistochemistry staining for GPX4, NRF2, and SLC7A11 in GC tissues and paracancerous tissues, which is expressed in the nucleoplasm or cytoplasm (original magnification: 200×). Case 1: GPX4 (A, B); case 2: NRF2 (C, D); and case 3: SLC7A11 (E, F). GPX4, glutathione peroxidase 4; NRF2, nuclear factor erythroid 2-related factor 2; SLC7A11, solute carrier family 7 member 11.

Immunohistochemistry was performed in 42 GC tissues and paracancerous tissues to examine the expression of GPX4, NRF2, and SLC7A11. Gastric cancer tissues showed more positive staining with GPX4 (Figure 2A and  2B), NRF2 (Figure 2C and  2D), and SLC7A11 (Figure 2E and  2F) when compared with adjacent non-tumor tissues; GPX4 and NRF2 staining was mainly seen in the nucleoplasm or cytoplasm, while SLC7A11 staining patterns were mainly cytoplasmic. Of all cases examined, 7 (16.67%) specimens of cancer tissues and 0 (0.00%) specimens of paracancerous tissues showed high expression of GPX4, 23 (54.76%) specimens of cancer tissues and 2 (4.76%) specimens of paracancerous tissues demonstrated high expression of NRF2, and 4 (9.52%) specimens of cancer tissues and 1 (2.38%) specimen of paracancerous tissues showed high-expression levels of SLC7A11. The high-expression rates of GPX4 (Pearson *χ* ^2^, *P* = .012) and NRF2 (Pearson *χ* ^2^, *P* < .001) were clearly higher than that of paracancerous tissues and were statistically significant (Table 2).

### Comparison of Expression Levels of Glutathione Peroxidase 4, Nuclear Factor Erythroid 2-Related Factor 2, and Solute Carrier Family 7 Member 11 in Different Cell Lines

**Figure 3.**
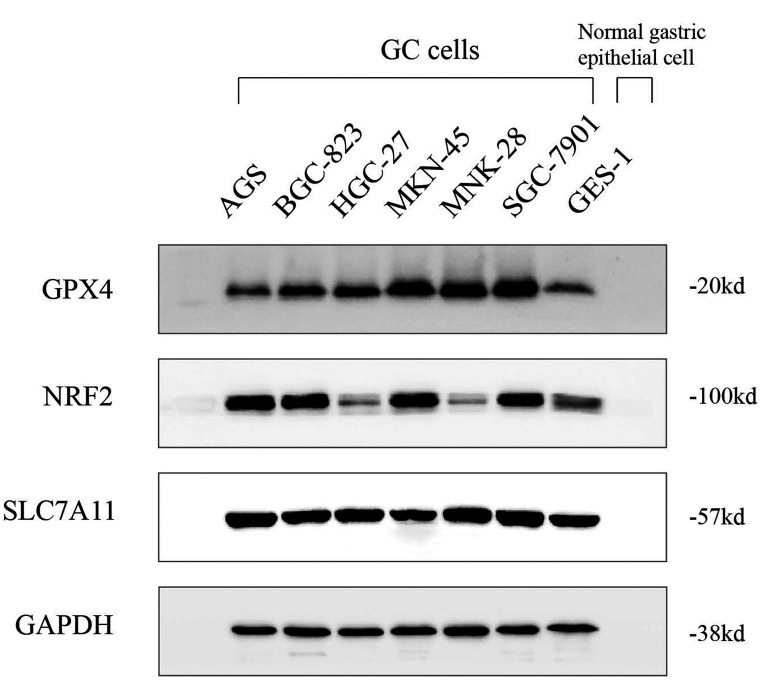
Expression of GPX4, NRF2, and SLC7A11 in 6 GCcell lines and GES-1 cell by western blot. GC, gastric cancer; GPX4, glutathione peroxidase 4; NRF2, nuclear factor erythroid 2-related factor 2; SLC7A11, solute carrier family 7 member 11.

**Figure 4.**
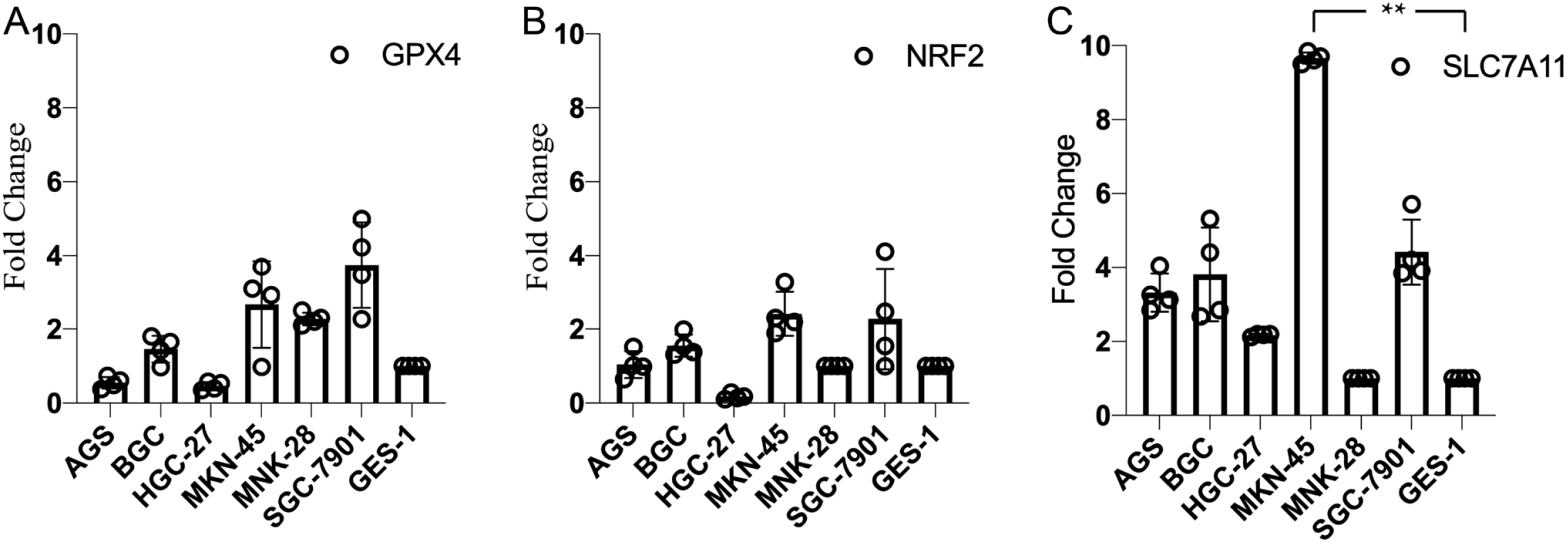
The mRNA level of GPX4 (A), NRF2 (B), and SLC7A11 (C) detected by RT-qPCR (^**^P < .01). GPX4, glutathione peroxidase 4; NRF2, nuclear factor erythroid 2-related factor 2; RT-qPCR, real-time quantitative polymerase chain reaction; SLC7A11, solute carrier family 7 member 11.

The cells were stably cultured and subcultured more than 3 times, and cellular morphology was recorded and distinguished well between cell lines. Glutathione peroxidase 4, NRF2, and SLC7A11 expression in 6 GC cell lines (AGS, BGC-823, HGC-27, MKN-45, MNK-28, and SGC-7901) and normal gastric epithelial cell GES-1 were detected by western blot and RT-qPCR after stable culture. Immunoblotting staining showed that GPX4 was more abundant in 5 GC cells except AGS relative to GES-1. Gastric cancer cells including AGS, BGC-823, MKN-45, and SGC-7901 exhibited high levels of NRF2 expression, while no significant difference was obtained between HGC-27, MNK-28, and normal gastric epithelial cells (Figure 3). In addition, qPCR further confirmed the results of GPX4 and NRF2 from western blotting (Figure 4). Surprisingly, GC cell line MKN-45 displayed a significant higher expression of SLC7A11 compared with that in GES-1.

### Association of Protein Expression and Clinicopathological Characteristics

Data regarding each protein expression and correlation about clinicopathological results are shown in Table 3. For purposes of analysis, tumor stages (stages I and II vs. stages III and IV) were assessed as dichotomized variables. There was no significant association between the expression of target proteins and sex, age, habits (smoking and alcohol), basic diseases (diabetes and hypertension), or tumor stage of patients (Table 3).

### Survival Analysis According to Protein Expression Incorporating Pathologic Features

**Figure 5.**
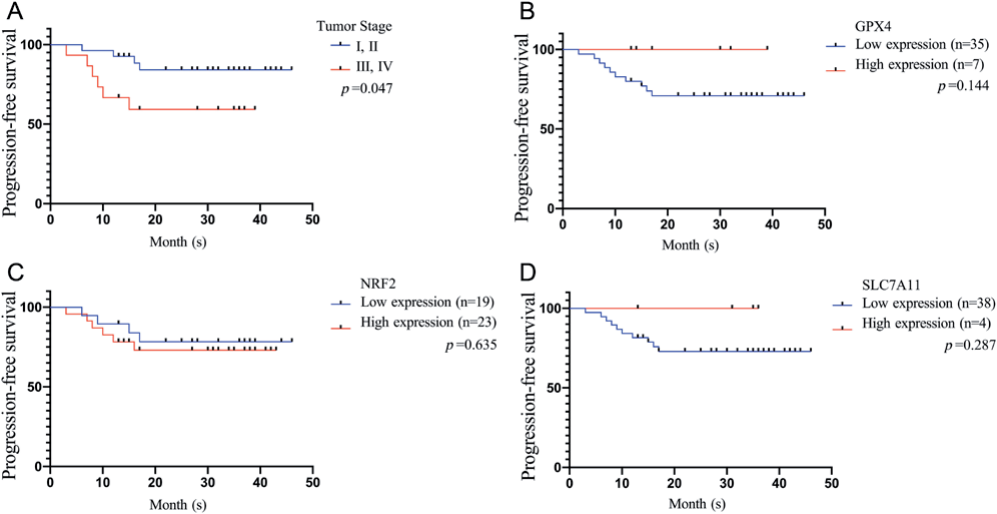
Kaplan–Meier (KM) survival curves of progression-free survival of 42 GC patients. The KM plot was used to detect the relationship between survival time and tumor stage (A), GPX4 expression (B), NRF2 expression (C), and SLC7A11 expression (D). GC, gastric cancer; GPX4, glutathione peroxidase 4; NRF2, nuclear factor erythroid 2-related factor 2; SLC7A11, solute carrier family 7 member 11.

To determine the association between GPX4, NRF2, and SLC7A11 with the prognosis of GC patients, disease outcomes of all patients were followed-up for 1 year or more after surgery. By the time when we conducted the analysis, 4 patients were dead; the time of death was 6 months, 7 months, 10 months, and 16 months after surgery respectively. Liver metastases occurred in 3 cases and were diagnosed at 3, 8, and 9 months postoperatively. One patient had developed bile duct metastasis, and the time of diagnosis of bile duct metastasis was 15 months after surgery. One patient had developed anastomotic carcinoma, and the time of diagnosis of anastomotic cancer was 12 months after operation. One patient had developed peritoneal carcinomatosis, and the time of diagnosis of peritoneal carcinomatosis was 17 months after surgery. Death or tumor progression (recurrence and/or metastasis) was regarded as the end point of analysis, and the progression-free survival (PFS) of all patients were calculated. When tumor stages (stages I and II vs. stages III and IV) were analyzed as dichotomized variables, there was significant difference between the PFS rate for patients and tumor stage of GC (*P* = .047, Figure 5A). Stratified according to protein expression, no significant difference was found between the PFS rate for patients and different expression levels of GPX4 (*P* = .144, Figure 5B) or NRF2 (*P* = .635, Figure 5C) or SLC7A11 (*P* = .287, Figure 5D).

### Database Analysis Based on Gene Expression Profiling Interactive Analysis

**Figure 6.**
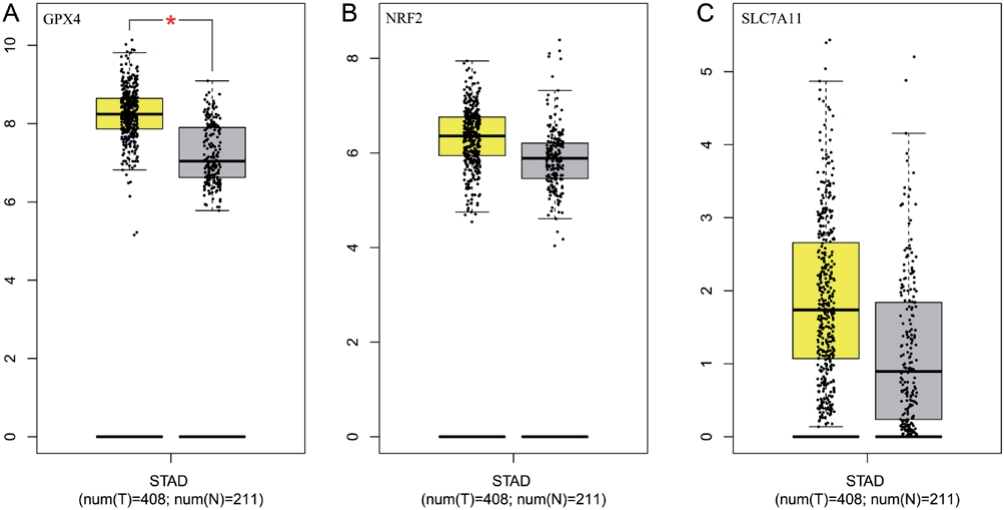
The expression of GPX4 (A), NRF2 (B), and SLC7A11 (C) mRNA in GC and normal gastric tissues with data from GEPIA. GC, gastric cancer; GEPIA, Gene Expression Profiling Interactive Analysis; GPX4, glutathione peroxidase 4; NRF2, nuclear factor erythroid 2-related factor 2; SLC7A11, solute carrier family 7 member 11.

**Figure 7.**
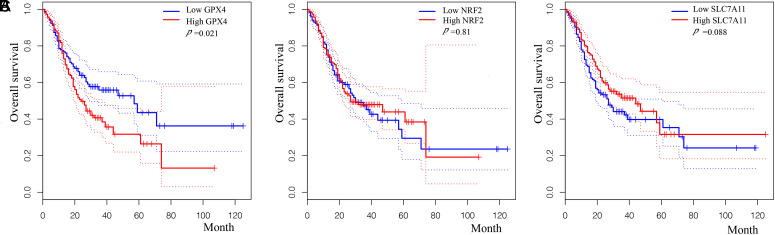
Kaplan–Meier (KM) analyses performed by GEPIA. The KM plot was used to detect the relationship between survival time and GPX4 expression (A), NRF2 expression (B), and SLC7A11 expression (C) respectively by GEPIA. GC, gastric cancer; GEPIA, Gene Expression Profiling Interactive Analysis; GPX4, glutathione peroxidase 4; NRF2, nuclear factor erythroid 2-related factor 2; SLC7A11, solute carrier family 7 member 11.

Analysis on transcription level was performed again by searching databases GEPIA. The expression of GPX4 (Figure 6A), NRF2 (Figure 6B), and SLC7A11 (Figure 6C) in GC was higher than that in normal tissues and varied in different stages of gastric tumor. Then, Kaplan–Meier analyses were again carried out to analyze the overall survival of patients by GEPIA. Glutathione peroxidase 4 expression was significantly correlated with the survival of GC (*P* = .021, Figure 7A), while the expression of NRF2 (*P* = .81, Figure 7B) or SLC7A11 (*P* = .088, Figure 7C) showed no trend toward patient survival.

## Discussion

Oxidative stress is likely the initiating factor for carcinogenesis, which is associated with the occurrence and development of various tumors including GC. A study of the digestive system showed that antioxidant enzyme GPX was related with the malignant phenotype in gastrointestinal cancers.^6^ Actually, GPX is a commonly used antioxidant marker in various gastric disease models, and among them GPX4 has the ability to resist lipid peroxides and protect cells from ferroptosis. Studies have detected a high-expression level of GPX4 in breast cancer,^7^ oral squamous cell cancer,^8^ and glioma.^9^ Its high expression was closely related with resistance to radiotherapy in non-small cell lung cancer. Glutathione peroxidase 4 also maintains the stem cell characteristics of pancreatic cancer^10^ and lung adenocarcinoma^11^ and promotes tumor proliferation, invasion, and metastasis. Wang et al^12^ showed that the mutation rate of GPX4 gene in GC was low (only 2.1%) by RNA sequencing of GC tissues from multiple tumor gene databases. In addition, in primary GC, GPX4-Mpr1/Pad1 N-terminal domain-containing protein (MPND) fusion gene can facilitate tumor growth and progression,^13^ which suggests that GPX4 may be a potential molecular marker for early diagnosis and prognosis of GC. Our study showed that GPX4 expression was significantly higher in GC tissues than in paracancerous tissues. Although some of the results showed that the high expression of GPX4 was significantly in connection with a poor prognosis by searching the GEPIA database, there was no significant relationship between the GPX4 expression in GC and patient survival in our current research, which might due to limited samples. On the contrary, Zhao et al^14^ reported that GPX4 was significantly downregulated in GC tissues compared to normal tissues, and low levels of GPX4 expression could be a risk factor for poor prognosis and survival in patients. A reasonable explanation for this finding was that the reduced expression of GPX4 may lead to a reduced ability to eliminate reactive oxygen species, subsequently resulting in increased proliferation, invasion, migration, and angiogenesis.

Nuclear factor erythroid 2-related factor 2 is a transcription factor that plays a major role in antioxidation, which is thought to be an important inhibitory factor of ferroptosis via regulating signaling including Nrf2–HO–1 axis and SQSTM1/p62–Keap1–Nrf2–AKR1C. High levels of NRF2 expression correlate with poor prognosis in lung tumor,^15^ gallbladder tumor,^16^ and diffuse large B-cell lymphoma.^17^ Here, we confirmed that NRF2 staining was concentrated in the nucleoplasm, and its expression was upregulated in GC compared to paracancerous tissues. Our results were in accordance with studies from Hafez et al.^18^ Furthermore, they found significant association between NRF2 expression and poor clinicopathological factors in GC, which was not verified in our study. Hu et al^19^ showed that NRF2 expression presented a prognostic significance in GC, and high expression of NRF2 was associated with poor patient prognosis. Together, high levels of NRF2 expression in GC lead to the production of antioxidants, which would enable these tumor cells to resist reactive oxygen species and gain higher malignant properties.^20^ These indicate that NRF2 expression levels may be used as a predictor of prognosis in patients with GC.

Reduced glutathione/glutathione disulfide is a main intracellular redox couple, and the level of glutathione is regulated by SLC7A11. It has been reported that SLC7A11 is highly expressed in some cancers, for example, lung cancer^21^ and colon cancer.^22^ A recent work had demonstrated that CD44-induced chemoresistance in GC could be attenuated by the inhibition of SLC7A11.^23^ In addition, Chinese herbal medicines such as Actinidia chinensis Planch^24^ and Tanshinone^25^ can inhibit the expression of SLC7A11 in GC cells, which finally aggravated ferroptosis and exerted antitumor effects. In the current study, we found no significant difference between SLC7A11 expression in GC and normal gastric tissues here, although we confirmed that GPX4 and NRF2 were highly expressed in GC. In recent years, ferroptosis has become a hotspot due to its potential antitumor effect. Previously, researchers noticed that the proliferation and malignant behaviors could be inhibited by inducing ferroptosis in HGC-27 and AGS cells.^26^ In fact, compared with paracancerous tissues, ferroptosis was significantly inhibited in GC cells, which is considered to promote tumor growth and reduce the sensitivity of cisplatin and paclitaxel chemotherapy.^27^ Current studies revealed some possible mechanisms of ferroptosis escape in GC, which involve the abnormal expression of cysteine dioxygenase 1,^28^ perilipin 2,^29^ stearoyl-CoA desaturase 1,^30^ and other factors. All these findings provided new insights for the diagnosis and treatment of GC. We expect this article will help researchers and clinicians to explore more potential markers of GC under the context of redox.

This study included a relatively limited number of clinical cases as the study was conducted in a single university affiliated hospital; these findings need to be evaluated in larger, multi-center prospective studies.

In conclusion, our study showed that GPX4 and NRF2 were upregulated in GC tissues compared to normal tissues. The high expression of GPX4 may be a marker of poor prognosis in GC patients. These results suggest that GPX4 and NRF2 may serve as potential novel biomarkers of GC patients clinically.

## Figures and Tables

**Table 1.** Forward Primers and Reverse Primers Used for RT-qPCR

Target Gene	Primer Sequences (5’→3’)	Size (bp)
GPX4-F	ACAAGAACGGCTGCGTGGTGAA	22
GPX4-R	GCCACACACTTGTGGAGCTAGA	22
NRF2-F	CACATCCAGTCAGAAACCAGTGG	23
NRF2-R	GGAATGTCTGCGCCAAAAGCTG	22
SLC7A11-F	TCCTGCTTTGGCTCCATGAACG	22
SLC7A11-R	AGAGGAGTGTGCTTGCGGACAT	22
GAPDH-F	GTCTCCTCTGACTTCAACAGCG	22
GAPDH-R	ACCACCCTGTTGCTGTAGCCAA	22

GPX4, glutathione peroxidase 4; NRF2, nuclear factor erythroid 2-related factor 2; RT-qPCR, real-time quantitative polymerase chain reaction; SLC7A11, solute carrier family 7 member 11.

**Table 2.** Expression of GPX4, NRF2, and SLC7A11 in Gastric Cancer and Paracancerous Tissues

Parameter	Cancer Tissues n (%)	Paracancerous Tissues n (%)	Pearson Correlation	*P*
GPX4 expression			0.302	.012^*^
High	7 (16.67)	0 (0.00)		
Low	35 (83.33)	42 (100.00)		
NRF2 expression			0.547	<.01^**^
High	23 (54.76)	2 (4.76)		
Low	19 (45.24)	40 (95.24)		
SLC7A11 expression			0.151	.356
High	4 (9.52)	1 (2.38)		
Low	38 (90.48)	41 (97.62)		

^*^P < .05.

^**^P < .01.

GPX4, glutathione peroxidase 4; NRF2, nuclear factor erythroid 2-related factor 2; SLC7A11, solute carrier family 7 member 11.

**Table 3.** Association of the Expression of GPX4, NRF2, and SLC7A11 with Clinicopathologic Characteristics of 42 GC Patients

Characteristic	Patients		GPX4 Expression				NRF2 Expression				SLC7A11 Expression			
			High		Pearson/Spearman	*P*	High		Pearson/Spearman	*P*	High		Pearson/Spearman	*P*
	n	%	n	%			n	%						
n	%
Total	42	100	7	16.67			23	54.76			4	9.52		
Gender					0.067	.666			–0.178	.337			0.073	.638
Male	27	64.29	5	18.52			13	48.15			3	11.11		
Female	15	35.71	2	13.33			10	66.67			1	6.67		
Age					0.067	.686			–0.121	.525			–0.073	.638
<60 years	15	35.71	3	20.00			7	46.67			1	6.67		
≥60 years	27	64.29	4	14.81			16	59.26			3	11.11		
Smoking					–0.239	.122			–0.128	.531			–0.102	.507
Yes	17	40.48	1	5.88			8	47.06			1	5.89		
No	25	59.52	6	24.00			15	60.00			3	12.00		
Alcohol					–0.141	.359			–0.166	.323			–0.205	.184
Yes	12	28.57	1	8.33			5	41.67			0	0.00		
No	30	71.43	6	20.00			18	60.00			4	13.33		
Diabetes					0.124	.421			0.066	.667			-0.090	.560
Yes	3	7.14	1	33.33			2	66.67			0	0.00		
No	39	92.86	6	15.38			21	53.85			4	10.26		
Hypertension					–0.003	.984			0.298	.053			0.134	.386
Yes	13	30.95	2	15.38			10	76.92			2	15.38		
No	29	69.05	5	17.24			13	44.83			2	6.90		
BMI					<0.001	1.000			0.039	.800			0.132	.391
<24	36	85.71	6	16.67			20	55.56			4	11.11		
≥24	6	14.29	1	16.67			3	50.00			0	0.00		
T					–0.250	.280			0.081	.457			–0.067	.896
1	7	16.67	0	0.00			3	42.86			1	14.29		
2	6	14.29	0	0.00			5	83.33			0	0.00		
3	24	57.14	6	25.00			13	54.17			2	8.33		
4	5	11.90	1	20.00			2	40.00			1	20.00		
N					–0.256	.499			0.147	.168			–0.140	.489
0	15	35.71	1	6.67			11	73.33			1	6.67		
1	13	30.95	2	15.38			4	30.77			1	7.69		
2	5	11.90	1	20.00			3	60.00			0	0.00		
3	9	21.43	3	33.33			5	55.56			2	22.22		
M					0.070	.651			–0.142	.358			0.051	.743
0 (absent)	41	97.62	7	17.07			22	53.66			4	9.76		
1 (present)	1	2.44	0	0.00			1	100.00			0	0.00		
Stage					–0.200	.195			–0.078	.611			–0.266	.085
I, II	27	64.29	3	11.11			14	51.85			1	3.70		
III, IV	15	35.71	4	26.67			9	60.00			3	20.00		
GPX4 expression									0.278	.071			–0.145	.347
High	7	16.67					6	85.71			0	0.00		
Low	35	83.33					17	48.57			4	11.43		
NRF2 expression					0.278	.071							–0.031	.841
High	23	54.76	6	26.09							2	8.70		
Low	19	45.24	1	5.26							2	10.53		
SLC7A11 expression					–0.145	.347			–0.031	.841				
High	4	9.52	0	0.00			2	50.00						
Low	38	90.48	7	18.42			21	55.26						

GC, gastric cancer; GPX4, glutathione peroxidase 4; NRF2, nuclear factor erythroid 2-related factor 2; SLC7A11, solute carrier family 7 member 11; T, tumor; N, node; M, metastasis.
